# 新一代基因测序技术及其在肿瘤研究中的应用

**DOI:** 10.3779/j.issn.1009-3419.2010.02.15

**Published:** 2010-02-20

**Authors:** 海粟 万

**Affiliations:** 300052 天津, 天津医科大学总医院, 天津市肺癌研究所, 天津市肺癌转移与肿瘤微环境实验室 Tianjin Key Laboratory of Lung Cancer Metastasis and Tumor Microenviroment, Tianjin Lung Cancer Institute, Tianjin Medical Univercity General Hospital, Tianjin 300052, China

基因测序技术的进步, 为分子生物学的发展, 起到了巨大的推动作用。传统的基因测序技术的重要代表, 是所谓的Sanger测序法, 这是一种以末端终止法为原理建立起来的技术^[1]^。20世纪90年代开始启动的人类基因组计划, 就是将Sanger测序法加以自动化的改进之后, 通过大规模的国际合作, 才最终完成的^[2]^。这项计划, 耗时十数年, 直接花费超过十亿美元。显然, 高昂的测序成本, 限制了基因测序技术的更常规的使用。近几年, 新一代基因测序技术(next-generation sequencing technology)的出现, 则呈几何级数地降低了基因测序的成本, 在不远的将来, 类似人类基因组规模的测序, 预期只需要1 000美元就可以完成^[3]^。

新一代基因测序技术, 由于测序成本的大幅度降低, 使之能够成为解决一般性的基因分子生物学问题的有效工具^[4, 5]^。新一代基因测序技术的发展, 无疑也为肿瘤分子生物学的研究, 提供了新的手段。本文将主要就新一代基因测序技术的特点和发展趋势及其在肿瘤研究中的应用, 作简要的综述。

## 1 新一代基因测序技术代表了生命科学技术发展的一种趋势

对于一个经典的生命科学实验, 其过程通常主要由三个部分组成, 即:实验材料和试剂的准备; 生物反应的进行, 对反应结果的观察和总结。将这一过程具体到传统的Sanger基因测序法, 即:序列片段和相关试剂的准备, 末端终止法测序反应的进行, 通过电泳将序列片段分离并观察结果。对这类经典的生命科学实验而言, 通常第一和第二两个步骤比较简单, 而最耗时耗力的是第三步, 对实验结果的观察。在传统的基因测序法中, 利用电泳将序列片段分离并观察结果, 是一个最影响测序效率的步骤。在人类基因组计划实施的过程中, 其所依赖的是对第三步实验过程的自动化。但即使这样, 人类基因组的成本依然很高。

显然, 要想从根本上降低生命科学实验的成本, 就必须大大降低以上对实验结果观察的费用, 而要从根本上降低序列片段的分离和观察的费用。新一代基因测序技术所代表的, 就是一个重要的方向:对实验反应的信号进行实时观察, 从而避免了以上所说的经典实验过程的第三个步骤的制约, 而也正是这个特点, 才使得测序成本能够被大大的降低^[6]^。

## 2 新一代基因测序技术的基本原理

严格地说, 所谓新一代基因测序技术, 并不是某种单一的技术, 而是一个技术群。不同的新一代基因测序技术, 在其原理上, 还是有很大的差别的。目前, 相对比较成熟、已经市场化或者接近市场化的, 主要有三家:Roche公司的454技术、Illumina公司的Solexa技术以及ABI公司的SOLiD技术。它们则分别使用了不同的测序原理。

### 2.1 454测序原理

454测序技术主要应用pyrosequencing原理:先使特异性的测序引物和单链DNA模板结合后, 在多种酶, 包括DNA聚合酶(DNA polymerase)、ATP硫酸化酶(ATP sulfurylase)、荧光素酶(luciferase)和双磷酸酶(apyrase)以及底物APS和Luciferin等的共同参与下, 将每一个dNTP的聚合与荧光信号的释放偶联起来。具体说来则是当向反应体系中加入1种dNTP, 如果它刚好能和DNA模板的下一个碱基配对, 则会在DNA聚合酶的作用下, 添加到测序引物的3’末端, 同时释放出一个分子的焦磷酸(PPi)。在ATP硫酸化酶的作用下, 生成的PPi可以和APS结合形成ATP; 在荧光素酶的催化下, 生成的ATP又可以和荧光素结合形成氧化荧光素, 同时产生可见光。通过CCD光学系统即可获得一个特异的检测峰, 峰值的高低则和相匹配的碱基数成正比。反应体系中剩余的dNTP和残留的少量ATP在Apyrase的作用下发生降解, 这样, 就可以在反应体系中, 加入另一种dNTP, 使以上反应重复进行, 根据获得的峰值图即可读取准确的DNA序列信息^[7]^(图 1)。

**1:**
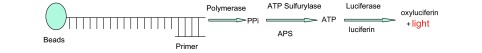
454测序原理示意图 Principle of 454 sequencing

### 2.2 Solexa测序原理

Solexa测序技术, 同样是利用DNA聚合酶的链延伸反应, 只是所使用的dNTP, 经过了特殊的修饰, 四种不同的dNTP分别被标记上了不同的荧光基团, 同时, 又在所有的dNTP中加入了3’末端保护基团, 即封闭基团, 以使每一步反应只能延伸一个碱基, 直到其荧光信号被收集后, 将荧光基团去除, 再将封闭基团去掉, 从而进行下一步反应, 而每步反应所收集到的荧光信号, 则对应了所要检测的序列。

在具体的实验操作中, 通常是在待测序列片段的两个末端加上含有特定序列的接头, 然后, 再利用专利的芯片进行:首先是待测序列在芯片上的原位扩增, 芯片表面含一层分别和待测片段两个末端的特定序列对应的两个单链引物, 可以通过互补原理, 捕获被变性成单链的待测片段, 引物扩增使得单链DNA成为双链, 该双链变性后成为单链, 其一端“固定”在芯片上, 另外一端(5’或3’)随机和附近的另外一个引物互补, 被“固定”住, 形成“桥”。这样的反应在上千万DNA单分子上发生, 形成的单链桥以周围的引物为扩增引物, 在芯片表面进行扩增, 形成双链。双链经变性成单链, 再次形成桥, 成为下一轮扩增的模板继续扩增, 经过30轮扩增, 每个单分子得到了若干倍扩增, 成为单克隆“DNA簇群”。其次, 是“DNA簇群”在Genome Analyzer综合分析仪上进行序列分析。如图 2所示, 正如前面所提及, Solexa测序技术采用“可逆性末端终止反应”进行的; 序列合成反应体系包括有引物、DNA聚合酶、4种标记了不同荧光的核苷酸, 每个核苷酸的碱基被保护基团封闭。每次反应掺入一个核苷酸, 该核苷酸类别可通过标记荧光进行识别, 经过扫描, 读取该次反应颜色后, 位于碱基3’末端的保护基团被除去, 继续下一轮反应, 如此反复, 得出片段的精确序列。该技术读长为30个-35个核苷酸^[8]^; 不过, 随着技术的不断改善, 其读长会逐步增加。

**2:**
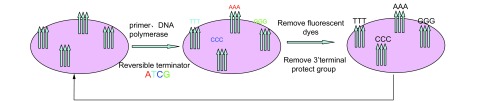
Solexa测序原理示意图 Principle of solexa sequencing

### 2.3 SOLiD测序原理

SOLiD应用连接法测序, 同时利用了独特的双碱基编码原理。待测的短的DNA片段的两侧, 被连上SOLiD接头, 分别是P1接头和P2接头, 然后, 则对加上接头的待测片段, 在特定的磁珠表面进行扩增, 具体则是通过油包水PCR反应进行的, 其中, 和P1接头对应的P1引物, 被固定在P1磁珠的表面, 在PCR反应前, 将含有PCR反应所有成分(其中包括P1磁珠和对应于P2接头的P2引物等)的水溶液, 注入高速旋转的矿物油表面, 水溶液就可以被分离成无数被矿物油包围的小液滴, 并各自构成独立的反应空间, 理想状况下, 每个小液滴只含一个DNA模板和一个P1磁珠, 随着PCR反应的进行, 磁珠上就形成了若干具有相同来源的扩增产物, 这就为后续的测序反应做好了准备。

如图 3所示, 测序反应, 是在前面制备的磁珠的基础上进行的; 先使用一个测序引物, 该测序引物与磁珠上的扩增产物的P1接头可以互补杂交, 而如果在其邻近的位置上, 存在另一个互补链, 则在测序引物和邻近的互补链之间, 可以进行连接反应; SOLiD系统使用了特殊的八个碱基长的寡核苷酸链, 其3’端第1、2位构成的碱基对是表征探针染料类型的编码区, 5’末端标有荧光染料, 因此不同的序列组合就被标记上不同的荧光基团。寡核苷酸链竞争性与测序引物邻近的序列杂交连接, 通过颜色判断序列组成, 当其标记颜色被读取后, 即将连接上的寡核苷酸在第五位和第六位之间切断, 以移除标记, 进行下一轮反应, 依此循环。在第一轮反应中, 可以得到确定的碱基位点为:1、2、6、7、11、12位碱基等。重复该反应过程, 偏移一位碱基, 使用较第一轮少一个碱基的引物进行反应, 如此往复, 直至整个序列读序完成。此技术目前的读长为30个-35个碱基^[9]^。

**3:**
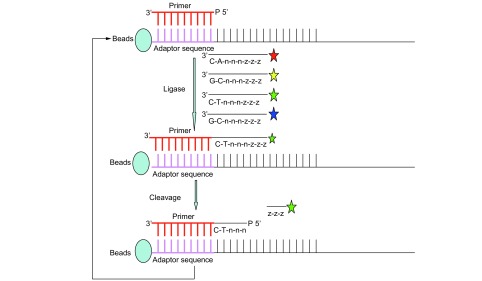
SOLiD测序原理示意图 Principle of SOLID sequencing

以上可见, 虽然这几种测序技术在基本原理上有所不同, 但都具有一个共同之处, 即都使用了反应信号的实时阅读, 在测序反应进行的同时, 就将反应信号收集起来。这样, 就几乎将经典的生命科学实验所需要的第三步省略了, 从而使得测序成本得到降低。

## 3 新一代基因测序技术的发展方向

以上所描述的, 是几种相对成熟的技术。但就人们对新一代基因测序技术的需求而言, 这几种技术, 还远谈不上完美。例如, 454技术的样品准备过程过于复杂, Solexa技术的测序阅读长度还比较短, 而SOLiD技术甚至还没有得到科研群体的广泛验证, 这些具体的问题, 都需要一一解决^[10]^, 但这些技术所面临的问题, 许多是其测序原理所固有的。事实上, 除这几种技术外, 许多依赖不同的原理的技术, 也正在研究中, 至少从目前看, 它们的潜力是非常巨大的。

例如:AQI Sciences公司推出有关荧光共振能量转移(fluorescent resonance energy transfer technologies, FRET)技术的系统平台。这种技术可以通过设计出相应的荧光标记核酸探针, 对靶DNA进行均相定性定量测定。对于长片段序列的测定, Agilent Laboratories公司也主要利用的是纳米孔道技术(nanopore technology)。纳米通道技术可以对基因快速测序, 并且能多组分(高通量)快速检测。最近, IBM公司改进了这种技术, 其方法是利用DNA晶体管来控制DNA在纳米孔道中的运动, 从而可以准确读出通过纳米孔道的碱基对, 达到精确测序的目的^[11]^。其它还有多家类似的从事高通量测序技术研究的公司或研究团队。例如:Helicos推出了一种单分子测序技术, 这种技术在测序时不再需要将基因组扩增成上千个拷贝, 而是只需要将一个拷贝的基因组打碎成30 bp左右的片段即可测序, 因此大大减少了测序的费用。这种技术已经被应用于临床检测。有研究^[12]^将这些更具潜力的基因测序技术, 称为新新一代基因测序技术(next next-generation sequencing technology)。在改善测序技术的同时, 人们同时也在关注如何更快更有效分析得到的大量数据^[13]^。例如, cDNA测序不仅确定表达水平, 而且确定等位基因特异性的基因表达。Kofler等^[14]^建立PanGEA方法, 就是利用454技术快速有效分析等位基因表达。PanGEA绘制454-est和基因或基因组对应图谱, 显示基因表达资料, 单核苷酸多态性以及等位基因特异性表达的定量化。Hackenberg等^[15]^建立了miRanalyzer—一种分析小分子RNA测序结果的工具, 可以用来检测miRBase中所有已知的microRNA序列, 找出所有最佳的转录序列, 并且预测新的microRNAs。

技术的总的发展趋势, 是更少的样品消耗、更高的通量、更长的阅读长度、更准确的测序结果、更低的成本^[16]^。相信在三到五年的时间内, 会有突破性的进展出现。

## 4 新一代基因测序技术为大规模高通量的研究提供了可能

新一代基因测序技术, 将许多从前无法实现的事情变成可能。如果说当年的人类基因组计划是一个巨大的挑战的话, 现在, 在新一代基因测序技术的基础上, 对不同物种基因组的测序, 几乎成为一个研究单位就可以决定的常规的行为^[17, 18]^。2009年初, 由中外科研单位共同提出的千人基因组计划, 也正式启动, 其总体目标, 是绘制一张关于人类基因多态性的高分辨率的图谱。

新一代基因测序技术, 其在生命科学领域的主要影响, 还在于使基因分子生物学的研究, 获得了一种大规模高通量的研究工具, 这得在全基因组水平上, 对组织细胞的分子机制的分析成为可能^[19-21]^。Zheng等^[22]^建立了多重的高通量管路以得到高质量数据, 并利用此方法对437个样本外显子的1 500个基因约5 Mb DNA重测序。

从对正常的发育过程的研究, 到对不同病理状况特征的研究, 研究人员都能获得更加全面数据^[23-25]^。这使得人类对生命现象的认识, 也从过去的单一过程的水平, 进入了整体的层次^[26, 27]^。

## 5 新一代基因测序技术在肿瘤研究中的应用

传统上的肿瘤分子生物学的研究, 开始是基本是对单一生物分子或者少数生物分子的分析, 后来随着技术的进步, 发展成为对单一分子过程或者少数分子过程的分析, 后来, 由于基因芯片的出现, 使得高通量集成化的分析成为可能, 而新一代基因测序技术, 则为肿瘤的分子生物学的研究, 提供了一种和基因芯片技术互为补充的新的高通量的工具^[28, 29]^。新一代基因测序技术, 对肿瘤分子生物学研究的影响是多方面的。

### 5.1 肿瘤基因组序列的再测序

高通量测序可以帮助研究者跨过文库构建这一实验步骤, 避免了亚克隆过程中引入的偏差。依靠后期强大的生物信息学分析能力, 对照一个参比基因组(reference genome)高通量测序技术可以非常轻松完成基因组重测序(re-sequencing)^[30-32]^。进而可以分析肿瘤基因组的拷贝数、多态性、不同类型的突变、基因相关性等^[33]^。

Gorlov等^[34]^利用高通量测序技术检测了基因编码区域上的83 715个SNP位点以确定肺癌的多态性敏感的变异体。在文中共分析了369例男性病例和287例对照例, 确定了22q12.2区域, 含有许多病例与对照不同的SNP位点, 这个结果与在细胞系中的实验结果相同。

### 5.2 全基因组基因表达谱的分析

Mortazavi等^[35]^人对小鼠的大脑、肝脏和骨骼肌进行了RNA深度测序, 这项工作展示了深度测序在转录组研究上的两大进展, 表达计数和序列分析。对测得的每条序列进行计数获得每个特定转录本的表达量, 是一种数码化的表达谱检测, 能检测到丰度非常低的转录本。分析测得的序列, 约90%的数据显示落在已知的外显子中, 同时, 也发现了许多序列并不在已知的外显子序列中, 而那些在已知序列之外的信息, 通过数据分析展示的是从未被报道过的RNA剪切(alternative splicing)、3’端非翻译区、变动的启动子(alternative promoter)以及潜在的小分子RNA前体, 发现至少有3 500个基因拥有不止一种剪切形式。而这些信息用传统技术是无法被发现的。

新一代高通量测序技术还被用于对基因转录起始位点的研究, 例如, Balwierz等^[36]^利用高通量测序技术分析了122个样本的转录起始位点, 构建了人和鼠的启动子转录起始位点图谱, 包括转录起始位点、转录起始簇和转录起始区域三个层次。同样, 这项研究也是很难用传统的方法实现的。

### 5.3 全基因组小分子RNA的分析

测序方法能轻易地解决芯片技术在检测小分子时遇到的技术难题(短序列, 高度同源), 而且小分子RNA的短序列正好配合了高通量测序的长度, 使得数据“不浪费”, 同时测序方法还能在实验中发现新的小分子RNA^[37, 38]^。

### 5.4 全基因组层次上甲基化分析

近年来研究者不断探索定性及定量检测单个或多个甲基化位点的方法, 但由于甲基化多态性区域存在的密度很高, 所以对于常规的延伸反应, 其引物的位置很难设计。焦磷酸测序技术能够快速地检测甲基化的频率, 对样品中的甲基化位点进行定性及定量检测^[39, 40]^。用焦磷酸测序技术检测基因甲基化水平, 常用重亚硫酸盐将基因组DNA中的未甲基化的胞嘧啶修饰为尿嘧啶, 甲基化的胞嘧啶则保持不变, 在以后的PCR扩增中, 尿嘧啶将变成胸腺嘧啶, 因此甲基化位点就成为一个普通的C/T单碱基多态性位点, 其中等位基因C的频率即为基因甲基化的程度, 这就可以通过测序的方法分析了。

White等^[41]^建立了针对Angelman综合征和praderwilli综合征*srnpn*基因甲基化检测平台, 诊断率达到100%。Shaw等^[42]^在甲基化特异性PCR(methylation specific PCR, MSP)基础上结合焦磷酸测序技术提出了甲基化强化焦磷酸测序技术(methylation enrichment pyrosequencing, MEP)改进了常规的焦磷酸测序甲基化分析(pyrosequencing methylation assay, PMA)对10例口腔鳞状细胞癌患者*p16*基因和细胞周期蛋白A1基因启动子进行甲基化分析, 结果显示MEP对甲基化位点的检出率明显比PMA为高, 与常规PMA不同的是, MSP引物是CpG位点特异性的, 从而提高了检测敏感度, 减少了假阳性结果。

Taylor等^[43]^利用454测序技术测序并分析40株细胞的25个CpG富集的基因, 发现ALL和FL样品的甲基化水平高于CLL和MCL, 并且, 在ALL和FL中甲基化从CpG岛的外周到中心递增散布。作者还通过同时分析基因组学和表观遗传学数据, 揭示了LRP1B启动子的单核苷酸多态性和甲基化水平之间的联系。

### 5.5 染色体结构的分析

在DNA-蛋白质相互作用的研究上, 染色质免疫沉淀-深度测序(ChIP-seq)实验也展示了其非常大的潜力^[44]^。染色质免疫沉淀以后的DNA直接进行测序, 对比ref seq可以直接获得蛋白与DNA结合的位点信息, 相比ChIP-chip, ChIP-seq可以检测更小的结合区段、未知的结合位点、结合位点内的突变情况和蛋白亲合力较低的区段^[45]^。和基因芯片一起, 新一代基因测序技术, 使人们能从整体的全基因组的层次上, 认识肿瘤的分子机制, 为肿瘤的预防和治疗, 提供了新的基础。

## 6 展望

如果说基因芯片技术是基因分子生物学研究领域的第一个高通量的研究技术的话, 新一代基因测序技术, 则同样构成了另一个重要的高通量的研究工具^[46]^。虽然还有许多问题需要解决, 但这项技术, 已经为基因分子生物学的研究, 带来新的变化, 而在肿瘤分子生物学的研究以及临床应用方面, 也显示了多方面的影响^[47, 48]^。相信随着测序通量的进一步提高和测序成本的进一步降低, 新一代基因测序技术, 将在肿瘤分子生物学领域, 发挥更加重要的作用。
